# The Association and Predictive Ability of ECG Abnormalities with Cardiovascular Diseases: A Prospective Analysis

**DOI:** 10.5334/gh.790

**Published:** 2020-09-01

**Authors:** Jingya Niu, Chanjuan Deng, Ruizhi Zheng, Min Xu, Jieli Lu, Tiange Wang, Zhiyun Zhao, Yuhong Chen, Shuangyuan Wang, Meng Dai, Yu Xu, Weiqing Wang, Guang Ning, Yufang Bi, Mian Li

**Affiliations:** 1 Department of Endocrine and Metabolic Diseases, Shanghai Institute of Endocrine and Metabolic Diseases, Ruijin Hospital, Shanghai Jiao Tong University School of Medicine, Shanghai, CN; 2 Shanghai National Clinical Research Center for Metabolic Diseases, Key Laboratory for Endocrine and Metabolic Diseases of the National Health Commission of the PR China, Shanghai National Center for Translational Medicine, Ruijin Hospital, Shanghai Jiao Tong University School of Medicine, Shanghai, CN

**Keywords:** Electrocardiography, Cardiovascular disease risk prediction, Reclassification, Discrimination, Calibration

## Abstract

**Aims:**

To examine whether electrocardiography (ECG) could provide additional values to the traditional risk factors for cardiovascular disease (CVD) risk prediction among different cardiovascular risk subgroups.

**Methods:**

A total of 7,872 community residents aged ≥40 years were followed up for a median of 4.5 years. A 12-lead resting ECG was examined for participants at baseline. CVD events including myocardial infarction, stroke and cardiovascular mortality were collected. Cox proportional hazards models were used and models of traditional risk factors with and without ECG were compared.

**Results:**

At baseline, 2,470 participants (31.3%) had ECG abnormalities. During follow-up, 464 participants developed CVD events. ECG abnormalities were associated with an increased risk of CVD after adjustment for the traditional risk factors in participants with a 10-year atherosclerotic CVD (ASCVD) risk ≥10% (hazard ratio, HR: 1.45; 95% confidence interval, CI: 1.11, 1.91). Adding ECG abnormalities to the traditional CVD risk factors improved reclassification for those who did not experience events [net reclassification index: 8.0% (95% CI: 2%, 19.5%)], discrimination (integrated discrimination improvement: 0.7% (95% CI: 0.1%, 1.9%), and calibration (goodness of fit P value from 0.600 to 0.873) in participants with a 10-year ASCVD risk ≥10%. However, no significant association and improvement were found in participants with a 10-year ASCVD risk <10%.

**Conclusions:**

ECG screening might provide a marginal improvement in CVD risk prediction in adults at high risk. However, ECG should not be recommended in adults at low risk.

## Introduction

Cardiovascular disease (CVD) is the most common cause of death worldwide and in China [1]. Because many patients do not have a diagnosis or symptoms of CVD before the first clinical event, identifying high-risk and asymptomatic individuals for early prevention and timely intervention is of great importance [2,3]. Risk prediction tools such as the Framingham Risk Score and the Pooled Cohort Equations use traditional CVD risk factors including sex, age, smoking, blood pressure, lipids, and diabetes status to calculate the probabilities of developing CVD events in the following 10 years [4,5]. Patients identified by these assessment tools as at high risk can benefit from preventive measures and improved risk assessments could lead to improved cardiovascular outcomes [6].

Electrocardiography (ECG), which is a commonly available, inexpensive, and noninvasive test, has been proved to be independently associated with CVD. ECG results might be a good candidate for cardiovascular risk stratification in asymptomatic individuals given its low cost, widespread use, and safety [7,8]. However, the US Preventive Services Task Force (USPSTF) recently updated its recommendations on ECG and recommended against screening with resting or exercise ECG for the prevention of CVD in asymptomatic adults at low risk (10-year event risk of <10%) [9,10]. More evidence on how the addition of ECG to the traditional risk factors affects reclassification in asymptomatic adults with intermediate or high risk of CVD events (10-year event rate of ≥10%) is awaited because the current evidence is insufficient to in this population [9,11].

Therefore, we used data from a community-based population cohort to assess the predictive ability of ECG for CVD events beyond traditional cardiovascular risk factors among asymptomatic Chinese adults aged ≥40 years and stratified on baseline cardiovascular risks.

## Patients and Methods

### Study population

Participants were recruited from community residents living in the Jiading district, Shanghai, China. Between March and August 2010, 10,375 community residents living in the area and aged ≥40 years participated in a baseline examination focused on cardiometabolic health. During August 2014 and May 2015, participants were invited to take part in a follow-up examination for the development of CVD events including myocardial infarction (MI), stroke, and heart failure. The study design and data collection were described previously [12,13].

For the current analysis, we excluded 355 participants with a CVD history or with missing data on CVD history, 20 participants with pacemaker implantations, 659 participants with missing data on ECG at baseline, and 1469 participants with missing data on CVD outcomes at follow-up. Therefore, a total of 7,872 individuals were finally included in the current analysis.

### ECG measurement

ECGs were recorded according to a standard protocol with participants in the supine position using a 12-lead ECG machine (CAM14, GE, US). Computer-assigned Minnesota Code (MC) to electrocardiographs of each participant was used for categorization. Major or minor ECG abnormalities previously reported to be associated with an increased risk of CVD including possible or probable MI, Q-QS wave abnormalities, left ventricular hypertrophy, Wolff-Parkinson-White syndrome, bundle branch block or intraventricular block, atrial fibrillation or atrial flutter, and ST-T changes were used [8,14,21]. Definitions of these ECG abnormalities based on the Minnesota Code are presented in Table 1.

**Table 1:** The Minnesota codes of the electrocardiographic abnormalities.

Electrocardiographic abnormalities Possible myocardial infarction	Minnesota Codes

*Moderate Q/QS waves without ST-depression or T-wave inversion*	1-2-1 to 1-2-7 without 4-1, 4-2, 5-1, and 5-2
*Minor Q/QS waves with ST-depression or T-wave inversion*	1-2-8 or 1-3-1 to 1-3-6 with 4-1, 4-2, 5-1, or 5-2
**Probable myocardial infarction**	
*Major Q/QS waves*	1-1-1 to 1-1-7
*Moderate Q/QS waves with ST-depression or T-wave inversion*	1-2-1 to 1-2-7 with 4-1, 4-2, 5-1, or 5-2
**Q-QS wave abnormalities**	1-1-1 to 1-2-8
**Left ventricular hypertrophy**	3-1
**Wolff Parkinson White syndrome**	6-4
**Bundle branch block or intraventricular block**	7-1, 7-2, 7-4 or 7-8
**Atrial fibrillation or atrial flutter**	8-3
**ST-T changes**	
*ST-depression*	4-1 or 4-2
*T-wave inversion*	5-1 or 5-2
*Minor ST- codes*	4-3 or 4-4
*Minor T-wave codes*	5-3 or 5-4

### Outcome assessment

The outcome was the time to first occurrence of a composite of CVD events including nonfatal MI, fatal and nonfatal stroke, and coronary heart disease death. Information on cardiovascular outcomes and vital status was collected from the national insurance system and local death registries. Two physicians independently reviewed medical charts and verified each clinical event with discrepancies resolved through discussion. It was calculated as the time from the baseline visit until the occurrence of the first CVD event in participants who had the interested outcome, until death in participants who died or until the follow-up visit in participants who did not develop CVD events.

### Covariates

Covariates including sociodemographic variables (age, sex, education), history of chronic diseases and medications, and lifestyle habits (smoking, drinking, physical activity) were evaluated using a standard questionnaire administered during a face-to-face interview. Body height, weight, and waist circumference were measured according to a standard protocol and body-mass index (BMI) was calculated as body weight divided by body height squared (kg/m^2^). Blood pressure (BP) was measured 3 times consecutively with 1-min intervals between each measurement after at least 5-min rest in a seated position using a calibrated digital electronic device (OMRON Model HEM-752 FUZZY, Omron Company, Dalian, China). The average of 3 BP measurements was used for analysis. Blood samples were collected after an overnight fast for ≥10 hours and biochemical parameters including total cholesterol, low-density lipoprotein (LDL) cholesterol, high-density lipoprotein (HDL) cholesterol, and triglycerides were measured using an autoanalyzer (Modular E170; Roche, Basel, Switzerland). Diabetes was defined by a self-reported history of diagnosis and/or the usage of anti-diabetic medications. Hypertension was defined as a systolic BP ≥140 mmHg and/or a diastolic BP ≥90 mmHg and/or having antihypertensive medications within 2 weeks.

### Statistical analysis

Differences in proportions and means of variables between participants with or without ECG abnormalities at baseline were assessed using the χ^2^ test and the analysis of variance, respectively. Cox proportional hazard models were used to evaluate the association between ECG abnormalities and incident CVD risks adjusted for traditional risk factors in the overall study population and stratified by the baseline 10-year atherosclerotic CVD (ASCVD) risk <10% or ≥10%, which corresponds with the USPSTF recommendations in adults with low and high CVD risks to inform clinical decisions on CVD primary prevention. The baseline ASCVD risk was calculated by the Pooled Cohort Equations [5]. To assess the additive value of ECG abnormalities to traditional CVD risk factors, we used and compared two survival models: model A included traditional CVD risk factors such as age, sex, smoking, systolic BP, diabetes status, total cholesterol, and HDL cholesterol; and model B included all risk factors in model A plus ECG abnormalities. Assumptions for the Cox proportional hazards models were verified by Schoenfeld residuals. Interactions were also assessed between ECG abnormalities and age, sex (no statistically significant interactions found).

Several statistical measures according to recommendations for the assessment of novel markers were examined [15]. To measure discrimination, we calculated and compared the Harrell’s C-statistics of models A and B [16]. Given that C-statistics may underestimate the improvement in predictive abilities [17], we also calculated the net reclassification improvement (NRI) of ECG abnormalities beyond traditional risk factors by comparing the predicted five-year CVD risks calculated by the two Cox model A and B [18]. The categorical NRI was calculated with cutoff points of 5% and 10% risk of CVD events over five years, corresponding with the thresholds in the US Adult Treatment Panel III (ATP III) guidelines to define low (<10%), intermediate (10%–20%), and high (≥20%) risk categories over 10 years [19]. The NRI was estimated according to the method by Pencina defined as {([number of events reclassified higher – number of events reclassified lower]/number of events) – ([number of non-events reclassified lower – number of non-events reclassified higher]/number of non-events)}. The former half of the formula is the event-NRI and the latter half is the non-event NRI [18]. In addition, we estimated integrated discrimination improvement (IDI) defined as the average increase in predicted risk among cases plus the analogous average decrease among controls, afforded by information on ECG abnormalities [17]. Calibration was examined using an extended version of the Hosmer-Lemeshow goodness-of-fit test for survival data, wherein greater *P* values indicate better calibration [20]. The 95% confidence intervals (CI) of these measures were calculated using bootstrap sampling with 500 repetitions.

Statistical analyses were conducted using SAS version 9.4 (SAS Institute) and R version 3.5.2 (R Project for Statistical Computing, http://www.r-project.org). A *P* value of <0.05 was considered statistically significant.

## Results

### Baseline characteristics

Among the 7,872 participants, 62.2% were women; the mean (SD) age was 57.8 (9.4) years; 2,470 (31.3%) had ECG abnormalities; and 1,609 (21.1%) had a 10-year ASCVD risk ≥10% at baseline. Participants with ECG abnormalities were more likely to be older, to be a male, to smoke and drink currently, to have higher BP and HbA1c levels, and to have higher ASCVD risks, compared with those without any ECG abnormalities (Table 2).

**Table 2:** Baseline characteristics of participants with or without ECG abnormalities.

Characteristics	Total (N = 7872)	ECG abnormalities		

		No (N = 5402)	Yes (N = 2470)	*P* value

Age, years	57.8 ± 9.4	56.9 ± 9.1	59.7 ± 9.8	<0.001
Women, n (%)	4899 (62.2)	3477 (64.4)	1422 (57.6)	<0.001
Education, n (%)				<0.001
Illiteracy	962 (12.3)	557 (10.4)	405 (16.5)	
Primary	1864 (23.8)	1231 (22.9)	633 (25.7)	
Secondary	4726 (60.3)	3370 (62.7)	1356 (55.1)	
Post-secondary	285 (3.6)	220 (4.1)	65 (2.6)	
Currently smoking, n (%)	1612 (21.1)	1066 (20.4)	546 (22.7)	0.018
Currently drinking, n (%)	816 (10.7)	518 (9.9)	298 (12.4)	0.001
Physical activity, MET-mins/week				0.378
<600, n (%)	3142 (40.5)	2178 (40.9)	964 (39.5)	
600–1499, n (%)	2262 (29.1)	1553 (29.2)	709 (29.1)	
≥1500, n (%)	2361 (30.4)	1595 (29.9)	766 (31.4)	
Body mass index, kg/m^2^	24.9 ± 3.3	24.9 ± 3.3	25.0 ± 3.3	0.545
Systolic blood pressure, mmHg	141.0 ± 20.0	138.7 ± 19.3	145.8 ± 20.8	<0.001
Diastolic blood pressure, mmHg	82.6 ± 10.4	82.1 ± 10.2	83.8 ± 10.7	<0.001
HbA1c, %	5.8 ± 0.9	5.8 ± 0.9	5.9 ± 1.0	0.002
Total cholesterol, mg/dL	208.8 ± 38.7	204.9 ± 38.7	208.8 ± 38.7	0.057
LDL cholesterol, mg/dL	123.7 ± 34.8	123.7 ± 34.8	123.7 ± 30.9	0.707
HDL cholesterol, mg/dL	50.3 ± 11.6	50.3 ± 11.6	50.3 ± 11.6	0.004
ACEI use, n (%)	438 (5.6)	283 (5.2)	155 (6.3)	0.063
Hypertension, n (%)	4651 (59.1)	2950 (54.6)	1701 (68.9)	<0.001
Diabetes, n (%)	651 (8.3)	453 (8.4)	198 (8.0)	0.574
10-year ASCVD risk ≥10%, n (%)	1609 (21.1)	945 (18.1)	664 (27.7)	<0.001

Abbreviations: ECG, Electrocardiographic; MET, metabolic equivalent; HbA1c, glycated hemoglobin A1c; LDL, low-density lipoprotein; HDL, high-density lipoprotein; ACEI, angiotensin-converting enzyme inhibitors; ASCVD, atherosclerotic cardiovascular diseases. Data are means ± SD for continuous variables and numbers (percentages) for categorical variables.

### Association of ECG abnormalities with CVD events

During a median follow-up of 4.5 years, 412 participants developed CVD events and 52 participants died of CVD causes. The incidence rate of CVD events was 17.1 per 1,000 person-years in participants with ECG abnormalities, compared with 10.9 per 1,000 person-years in participants without any ECG abnormalities (Table 3). After adjustment for age, sex, total cholesterol, HDL cholesterol, systolic BP, smoking, and diabetes, hazard ratios (HRs) and 95% CIs for CVD events in participants with ECG abnormalities vs. participants without ECG abnormalities was 1.25 (1.03, 1.51). Stratification of analysis by ASCVD risks at baseline revealed that ECG abnormalities were significantly associated with risks of developing CVD events in participants with an ASCVD risk ≥10% (HR: 1.45; 95% CI: 1.11, 1.91) but not in participants with an ASCVD risk <10% (HR: 1.09; 95% CI: 0.83, 1.44). Results were similar when education, BMI, LDL cholesterol, HbA1c, drinking status, physical activity, angiotensin-converting enzyme inhibitors usage were further adjusted. The associations between ECG abnormalities and individual CVD endpoints went in the same direction as the composite endpoint and were statistically significant in participants with an ASCVD risk ≥10%.

**Table 3:** Associations of ECG abnormalities at baseline with the development of cardiovascular events during follow-up.

	Without ECG abnormalities		With ECG abnormalities		Hazard Ratio (95% CI)	

	Events (n, %)	Incidence rate per 1000 person-years (95% CI)	Events (n, %)	Incidence rate per 1000 person-years (95% CI)	Adjusted for TCVRFs*	Adjusted for multivariables^†^

**Overall participants (n = 7872)**						
CVD	270 (5.0)	10.9 (9.7, 12.3)	194 (7.9)	17.1 (14.8, 19.7)	1.25 (1.03, 1.51)	1.25 (1.02, 1.53)
MI or CHD death	23 (0.4)	0.9 (0.6, 1.4)	29 (1.2)	2.5 (1.7, 3.6)	1.83 (1.04, 3.21)	2.06 (1.15, 3.70)
Stroke	248 (4.6)	10.0 (8.8, 11.3)	167 (6.8)	14.7 (12.6, 17.1)	1.19 (0.97, 1.46)	1.16 (0.95, 1.44)
**Participants with ASCVD risk ≥10% (n = 1609)^‡^**						
CVD	103 (10.9)	24.5 (20.2, 29.7)	113 (17.0)	38.1 (31.7, 45.8)	1.45 (1.11, 1.91)	1.45 (1.08, 1.95)
MI or CHD death	13 (1.4)	3.0 (1.7, 5.1)	21 (3.2)	6.7 (4.4, 10.3)	2.01 (1.01, 4.06)	2.29 (1.09, 4.81)
Stroke	91 (9.6)	21.6 (17.6, 26.5)	94 (14.2)	31.5 (25.7, 38.5)	1.39 (1.03, 1.86)	1.41 (1.04, 1.90)
**Participants with ASCVD risk <10% (n = 6007)^‡^**						
CVD	157 (3.7)	7.9 (6.8, 9.3)	79 (4.6)	9.8 (7.9, 12.2)	1.09 (0.83, 1.44)	1.10 (0.82, 1.48)
MI or CHD death	10 (0.2)	0.5 (0.3, 0.9)	8 (0.5)	1.0 (0.5, 2.0)	1.60 (0.62, 4.13)	1.84 (0.69, 4.88)
Stroke	147 (3.4)	7.4 (6.3, 8.7)	71 (4.1)	8.8 (7.0, 11.1)	1.06 (0.79, 1.41)	1.00 (0.75, 1.34)

Abbreviations: ECG, Electrocardiographic; CI, confidence interval; TCVRFs, traditional cardiovascular risk factors; MI, Myocardial infarction; CHD, coronary heart disease; ASCVD, atherosclerotic cardiovascular diseases. * Adjusted for traditional cardiovascular risk factors used to calculate the 10-year ASCVD risk score including age, sex, total cholesterol, high-density lipoprotein cholesterol, systolic blood pressure, smoking, and diabetes. ^†^ Adjusted for TCVRFs and education, drinking, physical activity, low-density lipoprotein cholesterol, BMI, HbA1c and ACEI use. ^‡^ Of the total participants, 256 participants with missing data on any of the traditional risk factors were not included in the separate analysis in different ASCVD risk subgroups.

### Additive value of ECG abnormalities

**Figure 1:**
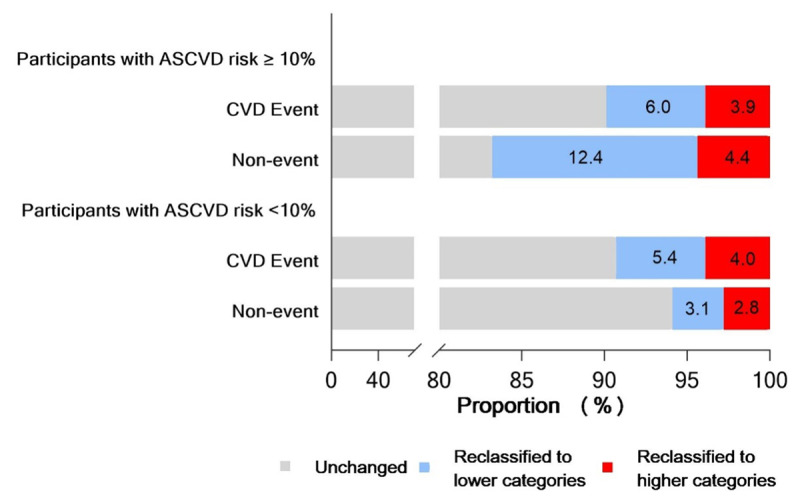
Reclassification of individuals by adding the ECG results to the ASCVD risk predicted model with traditional risk factors*. Abbreviations: ECG, Electrocardiography; HDL, high-density lipoprotein; ASCVD, atherosclerotic cardiovascular diseases; CVD, cardiovascular diseases. * The traditional risk factors included age, sex, smoking, systolic blood pressure, diabetes, total cholesterol and HDL cholesterol. Numbers are proportions of participants being reclassified.

Table 4 showed the predictive abilities of ECG abnormalities beyond traditional CVD risk factors for the development of CVD events. The C statistic improved from 0.699 (95% CI: 0.674, 0.723) to 0.701 (95% CI: 0.677, 0.725) with the addition of ECG abnormalities to traditional CVD risk factors in the overall participants and the improvement was more evident in participants with an ASCVD risk ≥10% (C statistics change: 0.014, *P* value = 0.06) compared with the improvement in participants with an ASCVD risk <10% (C statistics change: 0.004, *P* value = 0.47), although the changes in C statistics were not statistically significant. The calculated IDI was 0.2% (95% CI: 0.1%, 0.6%) in the overall participants. It was estimated to be 0.7% (95% CI: 0.1%, 1.9%) in high ASCVD risk participants and was close to 0% in low ASCVD risk participants. The categorical NRI indicated that there were an additional 8.0% (95% CI: 2%, 19.5%) of those who did not experience events were reclassified as low risk after adding ECG abnormalities to traditional risk factors and 2.0% of those who experienced events were incorrectly reclassified into a lower risk category in high ASCVD risk participants, although the net gain was not significant (6.0%, 95% CI: –2.2%, 13.2%). However, reclassification was not significantly improved in low ASCVD risk participants. Figure 1 shows the proportions of participants being reclassified.

**Table 4:** The predictive abilities of ECG abnormalities for the development of cardiovascular events.

Models	C statistic (95% CI)	C statistic change (95% CI)	IDI (95% CI)	NRI (95% CI)		

				Overall	Non-events	Events

Overall participants (n = 7616)*						
Model A	0.699 (0.674, 0.723)	0.002 (–0.003, 0.006)	0.002 (0.001, 0.006)	–0.031 (–0.044, 0.046)	0.007 (–0.004, 0.026)	–0.038 (–0.054, 0.036)
Model B	0.701 (0.677, 0.725)					
**Participants with ASCVD risk ≥10% (n = 1609)**						
Model A	0.587 (0.548, 0.626)	0.014 (–0.001, 0.035)	0.007 (0.001, 0.019)	0.060 (–0.022, 0.132)	0.080 (0.002, 0.195)	–0.020 (–0.096, 0.036)
Model B	0.601 (0.563, 0.639)					
**Participants with ASCVD risk <10% (n = 6007)**						
Model A	0.630 (0.595, 0.665)	0.004 (–0.007, 0.013)	0.000 (0.000, 0.001)	–0.012 (–0.054, 0.068)	0.002 (–0.008, 0.012)	–0.014 (–0.049, 0.065)
Model B	0.634 (0.599, 0.669)					

Abbreviations: ECG, Electrocardiographic; CI, confidence interval; IDI, integrated discrimination improvement; NRI, net reclassification index; ASCVD, atherosclerotic cardiovascular diseases. Model A used traditional CVD risk factors used to calculate the 10-year ASCVD risk score including age, sex, total cholesterol, high-density lipoprotein cholesterol, systolic blood pressure, smoking, and diabetes as predictors. Model B used traditional CVD risk factors in model A plus ECG abnormalities as predictors. * Participants with missing data on any of the traditional risk factors were excluded for the assessment of the additional value of ECG.

**Figure 2:**
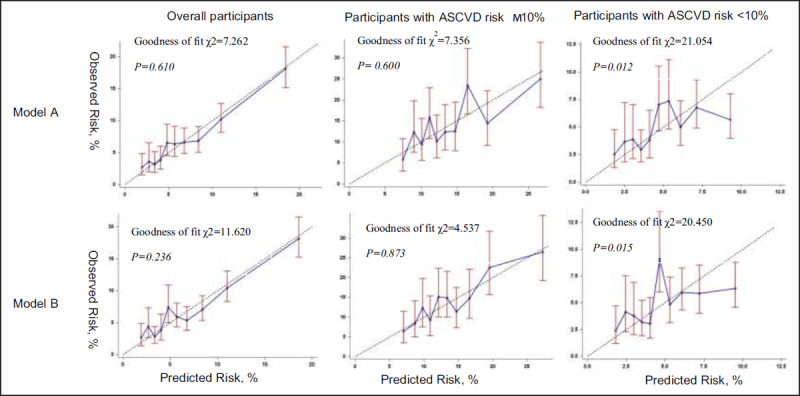
Calibration plots of the models with and without ECG abnormalities. Abbreviations: ASCVD, atherosclerotic cardiovascular diseases; ECG, Electrocardiographic. Data points indicate expected vs observed risk by deciles of predicted risk. The bars showed the 95% confidential interval of the observed risks. The dotted lines correspond to the lines of perfect calibration on which predicted risks coincide with the observed risks. Model A used traditional CVD risk factors used to calculate the Framingham Risk Score and the 10-year ASCVD risk score including age, sex, total cholesterol, high-density lipoprotein cholesterol, systolic blood pressure, smoking, and diabetes as predictors. Model B used traditional CVD risk factors in model A plus ECG abnormalities as predictors.

Figure 2 shows the calibration plots of the prediction models without and with ECG abnormalities. The CVD risk prediction model including traditional CVD risk factors has a good calibration (goodness of fit *P* = 0.610) in the overall participants and was not improved by the addition of ECG abnormalities (goodness of fit *P* = 0.236). When we classified the participants according to the ASCVD risk at baseline, the calibration of the prediction model in high ASCVD risk participants was improved when adding ECG abnormalities to traditional risk factors (goodness of fit *P* = 0.600 vs. 0.873). However, the calibration was not improved in low ASCVD risk participants (goodness of fit *P* = 0.012 vs. 0.015).

## Discussion

Using data from a well-defined community-based population cohort, we found that the resting ECG abnormalities were significantly and independently associated with an increased risk of developing CVD events and the addition of ECG abnormalities to the traditional CVD risk factors marginally improved the prediction of future CVD events in adults with a 10-year ASCVD risk ≥10%. However, the resting ECG abnormalities were not associated with an increased risk of developing CVD events and did not improve the prediction of future CVD events in adults with a 10-year ASCVD risk <10%. These findings were consistent with the USPSTF recommendations in that resting ECG should not be recommended as a screening tool to prevent CVD events in asymptomatic adults at low risk of CVD events. Whether ECG could be recommended for CVD prevention in adults at high risk of CVD events needs more evidence.

Although the USPSTF recommended against use of routine ECG as a screening tool to prevent CVD events in asymptomatic adults at low risk and concluded evidence insufficient in asymptomatic adults at intermediate or high risk [9], studies on how the addition of ECG to traditional risk factors affects CVD risk reclassification are limited. Several previous studies have reported similar associations between single and multiple ECG abnormalities and CVD outcomes, but few have examined the improvement in CVD risk discrimination, reclassification, and calibration by adding the overall CVD-related ECG abnormalities to traditional risk factors [8,21,22,23,24]. When examining the single ECG abnormalities, there were 946 (12.0%) left ventricular hypertrophies, 249 (3.2%) bundle or interventricular blocks, and 779 (9.9%) ST-T abnormalities and 333 (4.2%) possible myocardial infarctions in the current study, all of which were associated with increased risks of CVD events (adjusted HR: 1.12 to 1.54), although some of them were not statistically significant. The systematic review used for the USPSTF recommendations reviewed findings from nine cohort studies and showed that adding resting ECG to traditional risk factors resulted in small improvements in discrimination (absolute improvement in areas under the receiver operating characteristic curves or C-statistics: 0.001–0.05) for multiple cardiovascular outcomes, and total NRIs of adding resting ECG information ranged from 3.6% (2.7% for events and 0.6% for non-events) to 30% (17% for event and 19% non-events) [10]. In these studies, few have reported a statistically significant improvement and none has compared the predictive abilities of ECG between adults at low and high CVD risks [21,23]. Findings from the current study have added to the previous knowledge by demonstrating differences in associations and predictive abilities of ECG with future CVD risks between low and high CVD risk participants.

The inconsistencies between studies in reclassification might be due to the fact that the NRI is highly dependent on risk category thresholds, which vary widely across studies. Our study used the thresholds of 5% and 10% over five years in order to correspond with the thresholds used in the ATP III guidelines to define CVD risk categories [19]. In contrast to the NRI, the IDI is the integrated difference in Youden’s indices between models and therefore is not affected by choice of cutoff values [17]. However, few previous studies rarely reported IDI and its 95% confidence interval. The positive and significant absolute IDI in the high ASCVD risk participants found in the current study indicated that adding ECG to the traditional risk factors may improve the integrated discrimination of the prediction model.

Consistent with the recommendations of the USPSTF and several other US medical societies [25,26], our study found that ECG screening added little to the traditional risk factors for CVD risk prediction in a low risk Chinese population. The use of ECG screening in individuals with elevated CVD risks is more controversial [9,10,11]. One question is whether the prediction of CVD by ECG screening in individuals with elevated risks will lead to more intensive medical interventions and the potential harm of subsequent procedures or interventions resulting from abnormal findings by ECG screening should also be considered. In the current study, the reclassification improved by ECG in the high-risk population was found in those who did not develop a CVD event, which indicated that ECG screening could correctly reclassify more individuals with normal ECG as individuals who have less chances to develop CVD, therefore to reduce unnecessary interventions and their potential harms. Cost-effectiveness studies of ECG screening in the prediction and prevention of CVD events in a high-risk population will be needed.

Besides ECG, previous studies have addressed the ability of other nontraditional risk factors (e.g., the ankle-brachial index, high-sensitivity C-reactive protein [hsCRP], and coronary artery calcium [CAC] score) in improving existing models [27,28]. A systematic review conducted by the USPSTF reported that all the above nontraditional risk factors brought very small improvements in discrimination and reclassification to base models with traditional risk factors. The hsCRP level showed the smallest effect on risk prediction (C statistic change 0.0039, NRI 0.0152). The largest improvements in discrimination (C statistic change ranging from 0.018 to 0.144) and reclassification (NRI ranging from 0.084 to 0.35) were seen for CAC score. Although ECG improved the prediction of CVD less than CAC score, ECG is a widely available tool and could be used by providers in resource limited settings where coronary artery calcium might not be available.

This study has several strengths. It is among the first to examine the additive value of ECG abnormalities for CVD risk prediction in a Chinese population. The data was from a large and well-characterized cohort of middle-aged and elderly community residents. Further, several statistical measures according to recommendations for the assessment of novel markers were reported and all of them were adapted to the survival data.

Limitations of our study should be considered. First, automated ECG readings by a computer were used in the current study and potential misclassifications might exist. However, automated ECG readings were widely used in the clinical settings thus our findings have important clinical implications. Second, a median follow-up of 4.5 years of the present cohort could result in a limited number of CVD events and the evaluation of improved predictive abilities of ECG for future CVD events could be more valid with a longer follow-up duration.

In conclusion, resting ECG abnormalities were associated with increased risks of CVD events and the addition of ECG to the traditional risk factors marginally improved the discrimination, reclassification, and calibration of CVD risks in participants with a high ASCVD risk. However, no significant association and improvement were found in participants with a low ASCVD risk. Whether ECG should be used in routine screening of high CVD risk individuals should be further evaluated.
